# Visualisation of gene expression within the context of tissues using an X-ray computed tomography-based multimodal approach

**DOI:** 10.1038/s41598-024-58766-5

**Published:** 2024-04-12

**Authors:** Kristaps Kairišs, Natalia Sokolova, Lucie Zilova, Christina Schlagheck, Robert Reinhardt, Tilo Baumbach, Tomáš Faragó, Thomas van de Kamp, Joachim Wittbrodt, Venera Weinhardt

**Affiliations:** 1 Centre for Organismal Studies, 69120 Heidelberg, Germany; 2 HeiKa Graduate School On “Functional Materials”, Heidelberg, Germany; 3 Heidelberg International Biosciences Graduate School HBIGS, Heidelberg, Germany; 4 Institute for Photon Science and Synchrotron Radiation (IPS), Karlsruhe Institute of Technology (KIT), Eggenstein-Leopoldshafen, Germany; 5 Laboratory for Applications of Synchrotron Radiation (LAS), Karlsruhe Institute of Technology (KIT), Karlsruhe, Germany; 6 European Molecular Biology Laboratory, Heidelberg, Germany

**Keywords:** Imaging and sensing, X-ray tomography

## Abstract

The development of an organism is orchestrated by the spatial and temporal expression of genes. Accurate visualisation of gene expression patterns in the context of the surrounding tissues offers a glimpse into the mechanisms that drive morphogenesis. We developed correlative light-sheet fluorescence microscopy and X-ray computed tomography approach to map gene expression patterns to the whole organism`s 3D anatomy. We show that this multimodal approach is applicable to gene expression visualized by protein-specific antibodies and fluorescence RNA in situ hybridisation offering a detailed understanding of individual phenotypic variations in model organisms. Furthermore, the approach offers a unique possibility to identify tissues together with their 3D cellular and molecular composition in anatomically less-defined in vitro models, such as organoids. We anticipate that the visual and quantitative insights into the 3D distribution of gene expression within tissue architecture, by multimodal approach developed here, will be equally valuable for reference atlases of model organisms development, as well as for comprehensive screens, and morphogenesis studies of in vitro models.

## Introduction

In the fields of developmental and cell biology, the regional distribution of gene activity can provide insights into the critical processes of an organism’s development and mechanisms causing diseases. Visualisation of molecular mechanisms within their native context is often limited to sections and remains challenging in mm-scale tissues. To overcome these limitations, we introduce correlative light sheet microscopy and X-ray microtomography allowing us to achieve simultaneous localization of fluorescent markers and visualization of tissue architecture with micrometres spatial resolution in organisms and tissues of hundreds of micrometers in size.

Fluorescence microscopy, particularly light-sheet microscopy, is a powerful tool to study gene expression in whole organisms and tissues at high throughput and high spatial resolution^1–5^. Recent developments in the clearing of opaque tissues^6^ and novel geometries in light-sheet microscopy^7^ enable the visualisation of fluorescent proteins in several centimetres thick specimens. Particularly combinations of clearing and expansion microscopy have become promising tools for nanoscale-resolution imaging of whole vertebrates^8,9^. Although general fluorescent dyes such as nuclei and cytoplasm labels or NHS ester^10^ can help to portray tissue context, their limited penetration and the specificity of fluorescence microscopy to labelled tissues also lead to the inability to visualize the whole content of complex biological structures. Fluorescence-based approaches such as multiplexing approaches^6,11^, clearing^12^ and expansion^9^ remain limited due to complex and time-consuming sample preparation procedures.

To mitigate the loss of contextual information, optical tomography, that is bright-field contrast available in fluorescence microscopes, has been applied to visualize various organisms^13^, including in vivo imaging of zebrafish embryos^14^. However, while useful for aligning 4D information or providing general outlines of the sample, optical tomography does not provide sufficient differential contrast of tissues and lacks structural information on the tissue level. Several multimodal approaches have been suggested to overcome these limitations. Correlative light-sheet and electron microscopy (CLEM) has been used to target and visualize the ultrastructure of bone marrow in zebrafish^15,16^. As a result of the limited penetration depth of electron microscopy, CLEM is, however, difficult to perform on whole tissues or organisms.

Due to its high penetration depth, X-ray computed tomography is particularly suitable for anatomical imaging of whole organisms with high-throughput^17–20^. Based on absorption contrast agents, pan-resolution histology-like, also called histotomography volumes, are obtained in diverse specimens^21–23^, enabling phenotypic screens^24,25^ and analysis of pathologies^26,27^. The application of phase contrast in X-ray computed tomography has expanded to live specimens^28–33^, with potential to enable dynamic in vivo multi-modal studies. The increasing prevalence of table-top CT scanners, equipped with micro- and nanofocus X-ray tubes and other compact X-ray sources in universities, research labs, and museums, coupled with the advances in higher throughput microtomography stations at high flux imaging beamlines of synchrotron radiation facilities, has made imaging of biological samples a routine practice^34–39^.

Here, we show that without modification of typical light-sheet and X-ray microCT scanner, we can perform a multimodal analysis that combines fluorescent data with morphological X-ray data, which delivers pan-resolution 3D structural information on whole organisms. We present the precision of this multimodal acquisition and data fusion, and demonstrate its applicability to targeted protein and mRNA sequences. Furthermore, we show how the approach offers possibilities for quantitative analysis of the reorganisation of gene expression upon gene mutation in fish embryos and new insights into the morphogenesis of in vitro tissue models.

## Results

### Implementation of a multimodal pipeline

**Figure 1:**
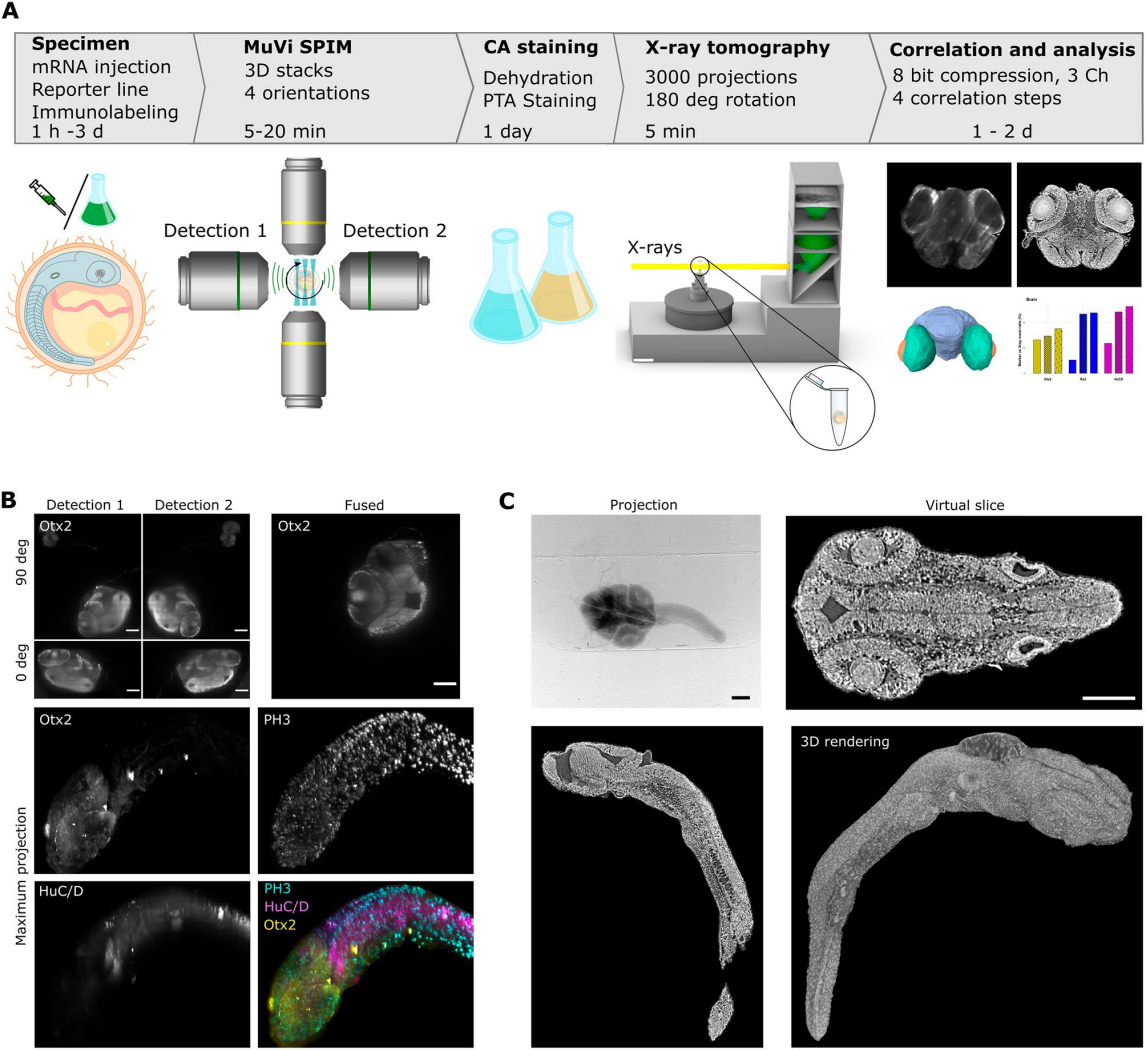
Correlation of light-sheet microscopy and X-ray microtomography into a multimodal pipeline. (**A**) Scheme representing main stages for the multimodal pipeline. Fluorescently tagged specimens are imaged with the multiview light-sheet microscope (MuVi SPIM), then dehydrated and stained with contrast agent (CA), here phosphotungstic acid for 1 day (depending on the specimen size), imaged with X-ray microtomography, followed by correlation of two modalities and analysis of gene expressions in different tissues. (**B**) Exemplary fluorescent images were obtained with the multiview light-sheet microscope for detection directions at 0 and 90 degrees rotation of the specimen, including the result of fusion. Maximum projection images from the MuVi SPIM of the medaka embryo (stage 24) immunolabelled with antibodies against Otx2, PH3 and HuC/D proteins. (**C**) X-ray projection image reconstructed virtual slices and 3D rendering of the same embryo as in panel (B) after staining with phototungstic acid. Scale bars are 100 µm.

To implement imaging of gene expression within tissue context, we took advantage of a multiview light-sheet microscope^4,5^ and X-ray absorption microtomography optimized for imaging of small model organisms^20,40^. Both imaging methods allow micrometer spatial resolution with short imaging times, enabling high-throughput generation of digital 3D atlases^18,22,41,42^. As the correlative approach relies on two separate instruments, we optimized sample handling, imaging parameters, and data analysis across a range of fluorescent labelling and gross morphology of samples (Fig. 1).

Fluorescently labelled specimens were imaged at two orthogonal orientations (0 and 90 degrees) with 2 detection planes at the multiview light sheet microscope (MuVi SPIM) (Luxendo, Light-sheet, Bruker Corporation). Multiview tomographic reconstructions resulted in an isotropic 3D volume with a voxel size of 406 nm and a total volume of about 830 µm × 830 µm × 830 µm (Fig. 1B). Due to the high penetration depth of hard X-rays, such volume can also be easily imaged with X-ray microtomography; however, it typically suffers from a lack of contrast^43,44^. Therefore, we employed chemical staining with phosphotungstic acid (PTA) to increase the absorption of hard X-rays of specimens as previously reported for different model organisms^20,22,40,45^. To implement PTA staining before X-ray imaging, instead of embedding specimens in low melting agarose, we put the specimens on the solid support of 2% agarose, which allows for the exchange of specimens in the MuVi SPIM microscope (Supplementary Fig. 1). This method facilitated the exchange of samples within a couple of minutes and ensured consistent specimen height positioning in the SPIM microscope. If an open-top SPIM microscope is available^46^, such embedding is not required, enabling imaging on a larger scale. For X-ray imaging, we have imaged PTA-stained specimens with broad energy bandwidth X-ray radiation, resulting in 70 ms exposure time for a single projection, 5 min time for complete specimen acquisition with the 3D volume of 2.5 mm × 2.5 mm × 2.5 mm and isotropic voxel size of 1.22 µm (Fig. 1C).

To build up the correlation pipeline, we made use of the image processing package Fiji^47^, mainly the tool for registration and alignment of 3D datasets—Fijiyama^48^. We selected this package for its adaptability, which permits to refine parameters and registration algorithms, enabling to establish a workflow more tailored to one’s specific needs. Of the two available registration algorithms, such as the iconic feature based^49^ and the block matching algorithms^50^, we chose the latter one. In general, all steps can be subdivided into three main parts (Supplementary Fig. 2: 1) compression of the original data to ease the handling of large datasets; 2) registration of X-ray microtomography to light-sheet datasets; 3) correlation of the original quality data and downstream analysis.

The compression of the original data serves two purposes: on the one side, to decrease the volume of the original datasets by cropping (X-ray tomography), rescaling to larger pixel size (light-sheet microscopy) and conversion of 16-bit (light-sheet microscopy) or 32-bit (X-ray tomography) to computationally less demanding 8-bit datasets. On the other side, fluorescent labelling can be sparse and low in its structural content (see Channel 3 in Supplementary Fig. 2). Thus, combining multiple channels into a single dataset improves registration accuracy. The designed compression steps allowed the downsizing of 72 Gb (light-sheet microscopy, 3 channels, 24 Gb each) and 30 Gb (X-ray tomography) to a few hundred Mb which could be handled without specialized machines for image analysis.

The registration of the datasets was performed at first manually to approximately overlay two datasets and then automatically for accurate registration, typically down to a single nucleus. Due to variable 3D orientation of samples within the SPIM, we chose manual registration as a first step. That was particularly necessary for organoids, which do not have stereotypic shape. For stereotypic specimens, like medaka or zebrafish, previous reports show that fully automatic registration can be used^51,52^. For manual registration, the use of gross specimen morphology and a few landmarks, such as eye lenses, otoliths, and tail in an embryo (Supplementary Fig. 3), provided a minimum of 5 datapoints to align light-sheet and X-ray volumes for further automatic registration. After registration, the obtained transformation parameters were applied to the original 32-bit X-ray microtomography data. Notably, performing transformation on an X-ray tomography dataset (and not on light-sheet data) allows to preserve high spatial resolution of light-sheet data and minimizes computational time compared to three channels in light-sheet microscopy. The final correlated multimodal datasets were used to perform segmentation for the digital anatomy of specimens and mapping of gene expression patterns throughout the whole tissue anatomy context (Fig. 1A and Supplementary Fig. 2). Overall, the multimodal approach requires antibody staining to be performed over 3 days, albeit several samples in parallel can be prepared. SPIM imaging including sample mounting takes roughly 15 min per sample, leading to a total imaging time of 7.5 h for a set of 30 samples. X-ray imaging is notably efficient, able to process 30 samples in about 1 h at the synchrotron radiation facility. The registration with 2–3 h of iterations per sample is the most time-consuming step. In sum, the complete protocol takes around 12 workdays for 30 samples chosen in this study.

### High precision in the visualisation of gene expression within the context of surrounding tissues

**Figure 2:**
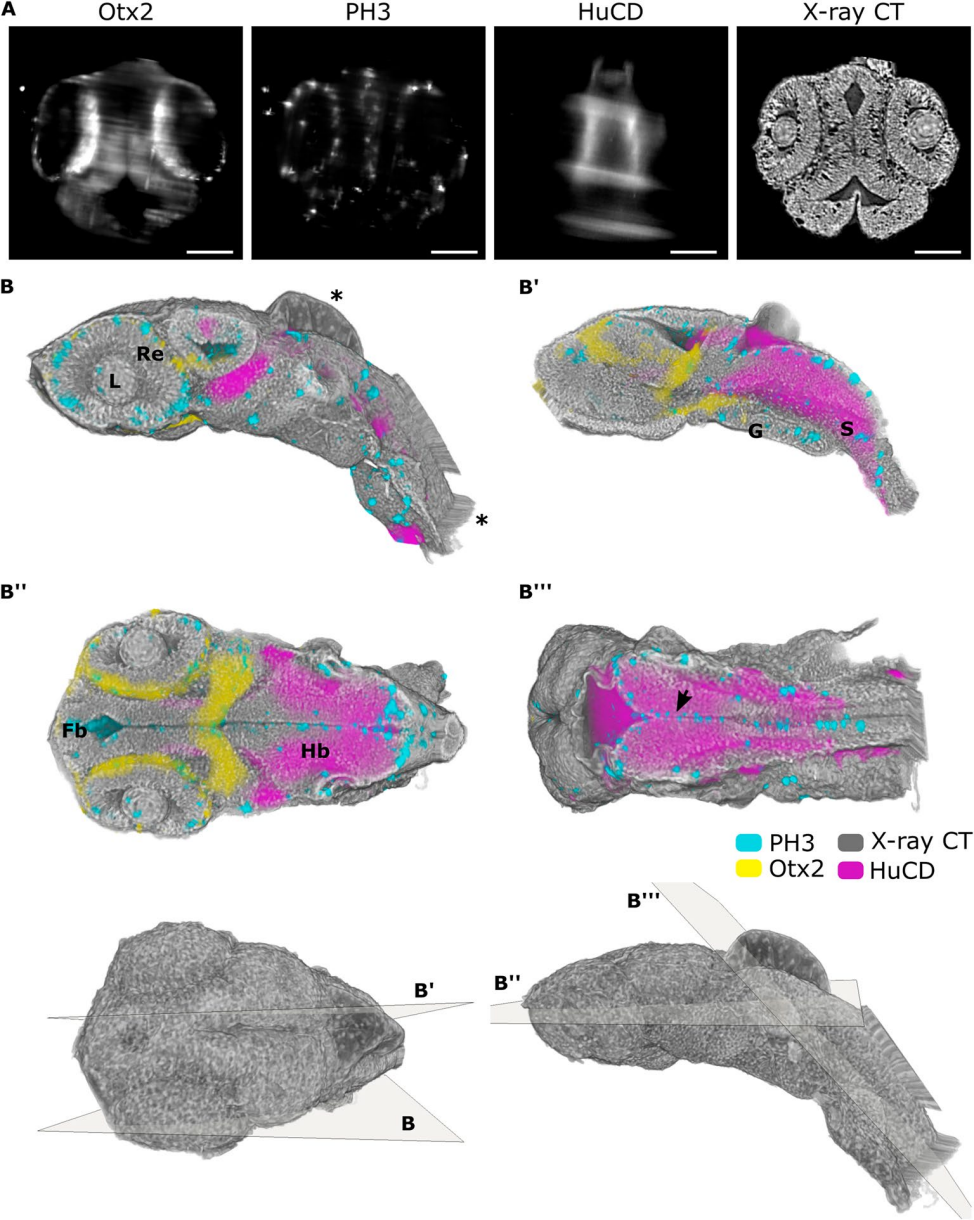
Neuronal genes in the context of tissue anatomy of the medaka embryo visualized by the multimodal approach. (**A**) Virtual sagittal slices of registered MuVi SPIM and X-ray tomography datasets. From left to right immunolabelling of Otx2, PH3 and HuC/D along with X-ray absorption in stage 24 of medaka embryo. (**B**) 3D renderings of all contrast modalities visualized with virtual cuts in axial, coronal and sagittal planes. The position and orientation of cuts is shown in the 3D rendering of the whole embryo. Misregistration and bulging of the head region due to dehydration is depicted with *. Organs are labelled as follows: lenses (L), retina (Re), forebrain (Fb), hindbrain (Hb), gut tube (G) and somite (S). Scale bars are 100 µm.

To assess the value of the multimodal imaging, we recorded and superimposed light-sheet and X-ray tomography volumes of stage 24 and 32 medaka embryos^53^. Making use of all available filters in the MuVi SPIM microscope, we visualized mitotic cells by anti-phosphorylated histone H3 marker (PH3), newborn neurons using the pan-neuronal marker HuC/D^54–56^ and forebrain using marker for Orthodenticle homeobox 2 (Otx2)^57,58^. The correlation of two imaging modalities showing high precision, particularly in the head and the eye regions, is demonstrated by transverse, sagittal, and coronal cross-sections at 1.22 µm^3^ isotropic voxel resolution (see Fig. 2A and B, Video 1). The accuracy of registration between X-ray and SPIM datasets was assessed on three coronal sections by checkerboard visualisation, demonstrating successful alignment (Supplementary Fig. 4). The regions at the border of the imaged volume, such as a tail, show artefacts of the registration with the unfaithful position of markers (Fig. 2B). Besides, due to dehydration steps applied during the staining procedure for X-ray tomography, the epithelium above the hindbrain was bulging in all datasets (Fig. 2). Based on the registration, we found that the dehydration step required for PTA staining results in 20.5 ± 9.5% (n = 5) shrinkage of specimens in X-ray tomography compared to light-sheet microscopy. Nevertheless, cellular contrast of X-ray tomography reveals a stereotypic spatial patterning of complex organs such as the developing retina, brain, somites and the developed gut tube along it (Fig. 2A, B–B′′′). At the outermost layer of the early retina, a single layer of retinal pigment epithelium precursors marked by Otx2 signal in SPIM can be distinguished by flat cell morphology typical for this cell type in X-ray tomography (Fig. 2B′′ and zoom in Supplementary Fig. 5). For both stages, that is 24 and 32, Otx2 expression is visible in retinal pigmented epithelium and in progenitors or differentiated photoreceptor cells of the retina (Fig. 2, Supplementary Fig. 6).

Otx2 expression is also found in the choroid plexus and the most posterior part of the mesencephalon at stage 24 (Fig. 2B′′, Video 1) with further specification in the hypothalamus and a scattered cell population in the optic tectum by stage 32 (Supplementary Fig. 6B′′′′). The newborn neurons labelled by HuC/D expression are located in the hindbrain and the neuronal tube (Fig. 2B′′′). Several studies have reported that dividing cells in the retina and brain migrate apically towards the periphery of the tissue^59,60^. Correspondingly, the multimodal imaging shows the localisation of mitotic cells at the outer part of the retina and at the interfaces of the hindbrain and somites which are regions unconstrained by any surrounding tissue (Fig. 2B, B′′′ and zoom in Supplementary Fig. 5).

Due to the difference in contrast between two imaging modalities, X-ray tomography provides information with content beyond the generalized labelling of nuclei or cell cytoplasm. The difference in contrast is demonstrated by the representative sagittal and coronal cross sections of a stage 24 medaka embryo stained with DAPI and N-cadherin (Supplementary Fig. 7). Notably, X-rays are unique in the ability to visualize whole tissue structures. Furthermore, the difference in X-ray absorption due to varying density of nuclei and extracellular matrix in organs helps to differentiate individual tissues within organs, such as the brain and layers in the retina, enabling quantitative analysis of individual cells and tissues^20,22^.

### Targeted sequence visualization in combination with X-ray tomography

While visualization of gene expression via detection of gene product by protein-specific antibody labelling is telling, we further asked whether the same correlative approach could be applied to specimens after fluorescence RNA in situ hybridisation (ISH). ISH allows the labelling of selected DNA and RNA strands and, due to such sensitivity and versatility, becomes the most extensively used technique for gene expression analysis^61^. To facilitate the entry of RNA probes and thus increase their hybridization efficiency, samples undergo several pre-treatment steps. These include methanol treatment to strip membrane lipids and improve permeability^62^ and digestion with Proteinase K to remove nuclear, cytoplasmic and extracellular matrix proteins enhancing probe hybridization^63^. As RNA ISH is typically performed as an end-point analysis method^64^, little is known about the preservation of proteins and connective tissue, which are known to be enhanced by PTA staining used in X-ray absorption tomography^65^.

After labelling, followed by PTA staining we have observed a variability in X-ray contrast-enhancement of tissue across developmental stages of medaka fish (Supplementary Fig. 8). For stages 22 and 23 (Supplementary Fig. 8, panel A), the tissue preservation was comparable to the differential X-ray contrast observed for the X-ray tomography data after antibody staining, see Fig. 2. For developmental stages 28 and 33, with the required time of Proteinase K digestion of 20 min, in comparison to 7 min for earlier stages, the differential contrast of tissues is not observed and PTA staining is visible as a superficial layer (Supplementary Fig. 8, panel B). In this manner, X-ray tomography can potentially be used to analyze tissue preservation after RNA ISH and other sample preparation techniques.

**Figure 3:**
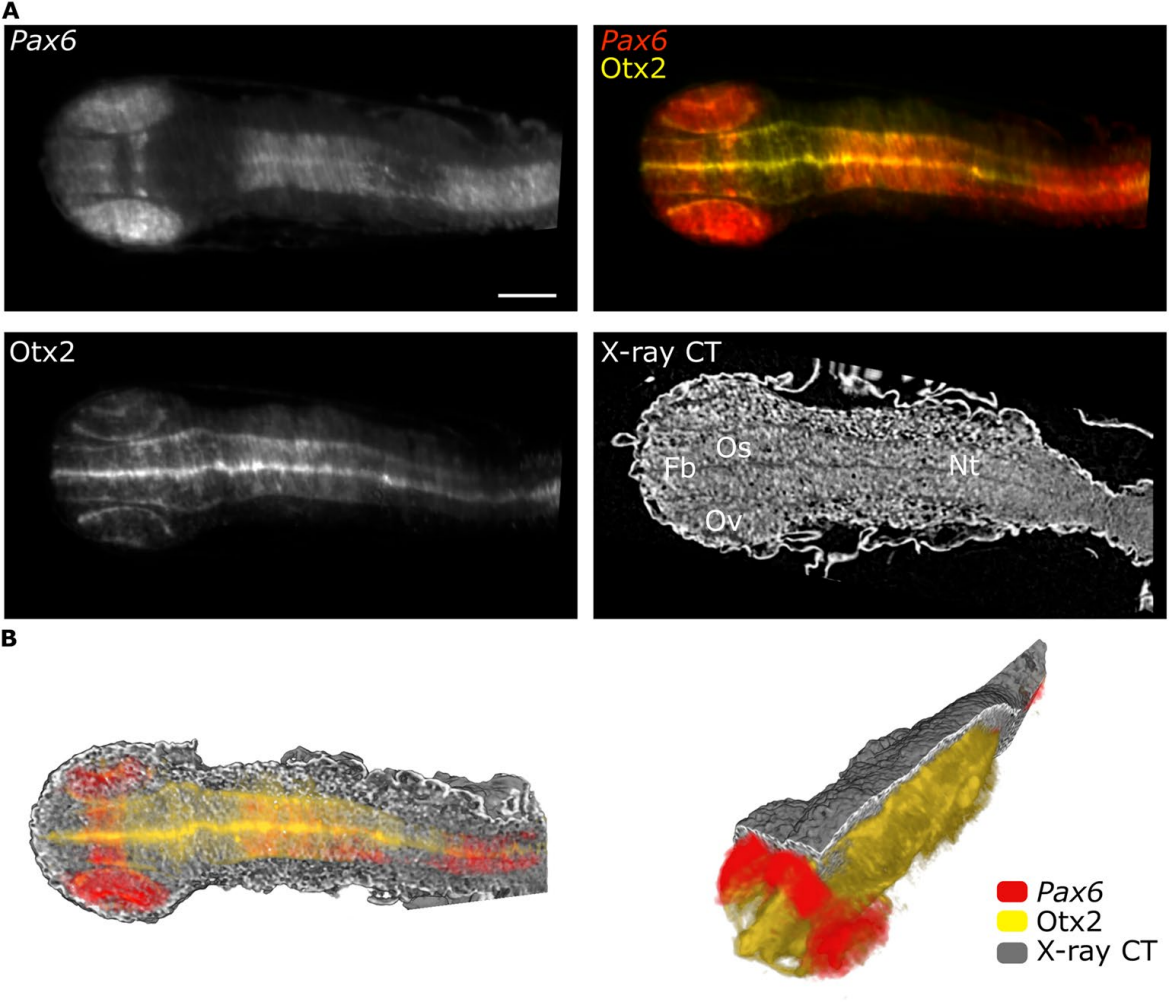
Targeted sequence labelling is correlated with X-ray tomography. (**A**) MuVi SPIM virtual slices of medaka embryo (stage 22) labelled by fluorescent mRNA in situ hybridisation, using *Pax6* mRNA antisense probe and immunolabelled with antibody against Otx2 protein. (**B**) 3D renderings of all contrast modalities with virtual cuts in transverse and sagittal planes. Organs are labelled as follows: optic vesicles (Ov), forebrain (Fb), optic stalk (Os) and neural tube (Nt). Scale bars are 100 µm.

For specimens with preserved tissue morphology after RNA ISH staining, we have used multimodal imaging to map the expression of key transcription factors for early eye and brain development Pax6 and Otx2. We have used simultaneous detection of *Pax6* mRNA (ENSORLG00000009913), using a *Pax6*-specific antisense mRNA probe from the medaka expression pattern database^66–68^, and antibody staining to detect expression of Otx2 protein on whole-mount medaka embryos as previously described for zebrafish embryos and larvae^69^. Consistent with previous reports^70,71^, *Pax6* transcripts were detected in the optic vesicles, in the forebrain at the site of the optic stalk and the neural tube at stage 22 of the medaka embryo (Fig. 3). At this stage, the expression of Otx2 is already apparent in the outer layer of the optic cup where the retinal pigmented epithelial is forming. X-ray tomography shows morphologically distinct populations of cells (Fig. 3). Accompanying optic vesicle evagination, two morphologically distinct populations of cells are visible in the retina and forebrain. The cells located at the end of the eye field are elongated and form a contiguous layer with the forebrain. The central part of the eye field is filled with cells of distinct morphology and cell density. This morphological transition might result from the neuroepithelial flow observed in teleost fish retina during early eye development^72^. Such visualization of cell patterning in relation to gene expression is possible due to the high spatial resolution and histology-like information of this multimodal approach.

### Quantitative analysis of phenotypic variations

**Figure 4:**
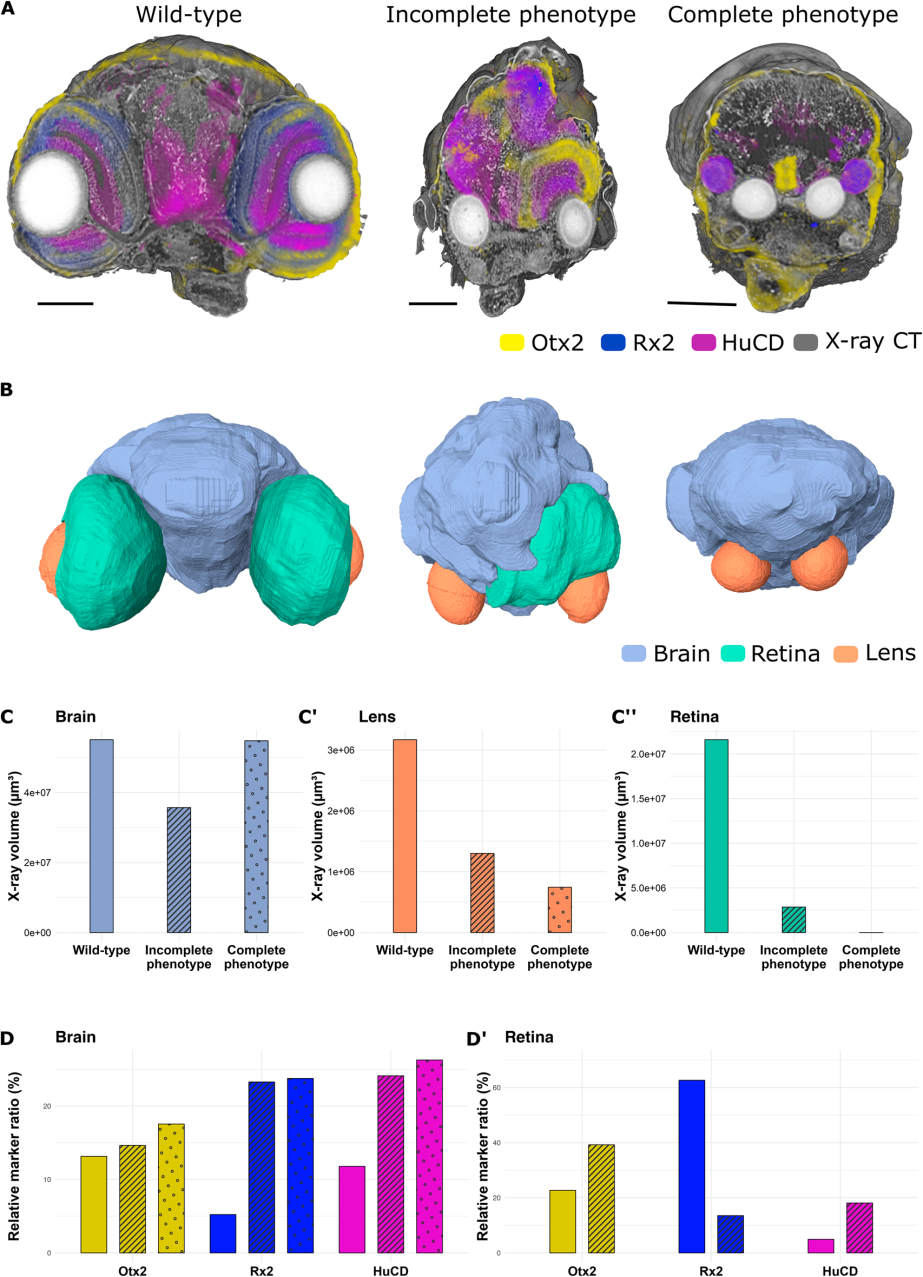
Quantitative analysis of phenotypic variations in *Rx3*^*saGFP*^ mutants. (**A**) Virtual slices through 3D rendering medaka embryo stage 34 labelled by whole-mount immunohistochemistry Otx2 (yellow), Rx2 (blue), and HuC/D (magenta) markers, imaged by multimodal approach. (**B**) 3D surface renderings of segmented based on X-ray tomography tissues, that is brain (blue), retina (green), and lens (orange). (**C**) Brain volume comparison across phenotypes revealed a decrease in the volume in the incomplete phenotype embryo compared with either wild-type sibling or complete phenotype embryo. (**C′**) Lens volume gets reduced as the phenotype gets more pronounced. (**C′′**) Retinal volumes along the severity of the phenotypes get smaller. (**D**)–(**D′**) Quantitative analysis of marker gene expression. In the brain, all the markers rise along the severity of phenotypes. In the retina, Otx2 and HuC/D get elevated in the incomplete phenotype while Rx2 gets smaller. Scale bars are 100 µm.

The multimodal approach can be particularly advantageous when studying morphological mutants. Previously, we reported the *Rx3*^*saGFP*^ mutant line, in which retinal progenitor cells in homozygous-mutant embryos fail to migrate laterally to form the optic vesicles (OV), thus mimicking the eyeless mutant^73–75^. The *Rx3*^*saGFP*^ homozygous mutants express continuous phenotypes, varying from smaller optic cups to the complete absence of eyes^75^. Taking advantage of such phenotypic differences and the histological information obtained from X-ray tomography, we asked how the gene expression patterns are altered in various retinal phenotypes. For that, we chose genes marking the main retinal compartments—Rx2 (retinal progenitor cells, ciliary marginal zone, Müller glia and photoreceptor cells), Otx2 (retinal pigmented epithelium, bipolar and photoreceptor cells) and HuC/D (amacrine and ganglion cells), see Fig. 4^55,75,76^. Medaka embryos of stage 34^53^ were first subjected to whole-mount immunohistochemistry to detect Rx2, HuC/D and Otx2 proteins and afterwards to X-ray tomography as described above. The resulting volumes were registered onto a combined correlated dataset (Fig. 4A). Using the X-ray tomography, we segmented retinas, brain and lenses in the embryo of every group: wild-type sibling (normal eye development), incomplete phenotype (altered optic vesicle evagination or altered optic cup morphogenesis) and complete phenotype (complete absence of the optic vesicle) (Fig. 4B). Based on the segmentation, we compared the volumes of each tissue between different phenotypes (Fig. 4C–C′′). The retina of the incomplete phenotype was much smaller than the retina of a wild-type sibling (Fig. 4C). One can also note that the retinal tissue is positioned between two lenses, forming a small optic cup on one side of the embryo (Fig. 4B, incomplete phenotype, retina in green). Such phenotype is caused by the altered migration of retinal progenitor cells from the developing forebrain reported previously^74^. The complete phenotype shows an example where such migration was fully arrested, resulting in the absence of optic vesicles (Fig. 4B, Complete phenotype). Interestingly, comparing brain volumes (segmented until the otic vesicle in all embryos), we could observe a decrease in the volume for the embryo with incomplete phenotype compared with either wild-type sibling or complete phenotype embryo (Fig. 4C′). Moreover, the lens volume decreases along with the increasing severity of the phenotype (Fig. 4C′′), suggesting the cross-talk between the retina and surface ectoderm which gives rise to the lens in embryonic development.

To investigate how the marker gene expression is altered between embryos of various phenotypes, we used automatic Otsu thresholding for all channels within combined labels of retina and brain, and obtained the voxel size occupied by either of the markers within the retina or brain. These values were then normalized by the size of the corresponding tissue based on the X-ray tomography (Fig. 4D–D′). Otx2 and HuC/D expression increased in the retina of the embryos with incomplete phenotype when compared to the wild-type sibling, while Rx2 expression drastically decreased. It is likely that proper differentiation of retinal cell types is affected in the incomplete phenotype due to the initially altered architecture of the developing optic cup. Thus, compartments marked uniquely by Rx2 (ciliary marginal zone and Müller glia) may be retinal progenitor cells, present to a lesser extent in the incomplete phenotype retina compared to the wild-type sibling. This would lead to the relative volume increase for the other two markers. In the brain, an increase in the relative ratio was observed for all three analysed markers (Fig. 4D′). This can be explained by the fact that, despite a migration arrest of retinal progenitor cells in the *Rx3*^*saGFP*^homozygous mutants, these cells were still able to obtain the retinal identity while located within the developing forebrain^75^.

Such quantitative analysis enabled by the multimodal approach allows for a systematic examination of phenotypic variations at a molecular level in mutants with unknown or continuous phenotypes, like in the case of *Rx3*^*saGFP*^ mutant.

### Multimodal characterisation of 3D organoid systems

The multimodal approach is particularly insightful for organisms that still need to be well described in terms of morphogenesis and 3D cell culture systems, such as explants and organoids. Organoids are valuable for modelling healthy or diseased human tissue and can be highly complex in cell composition^77,78^. To probe for the complexity of organoids concerning their in vivo counterparts, new 3D imaging approaches, which combine the identification of cell types with tissue morphology, are essential.

**Figure 5:**
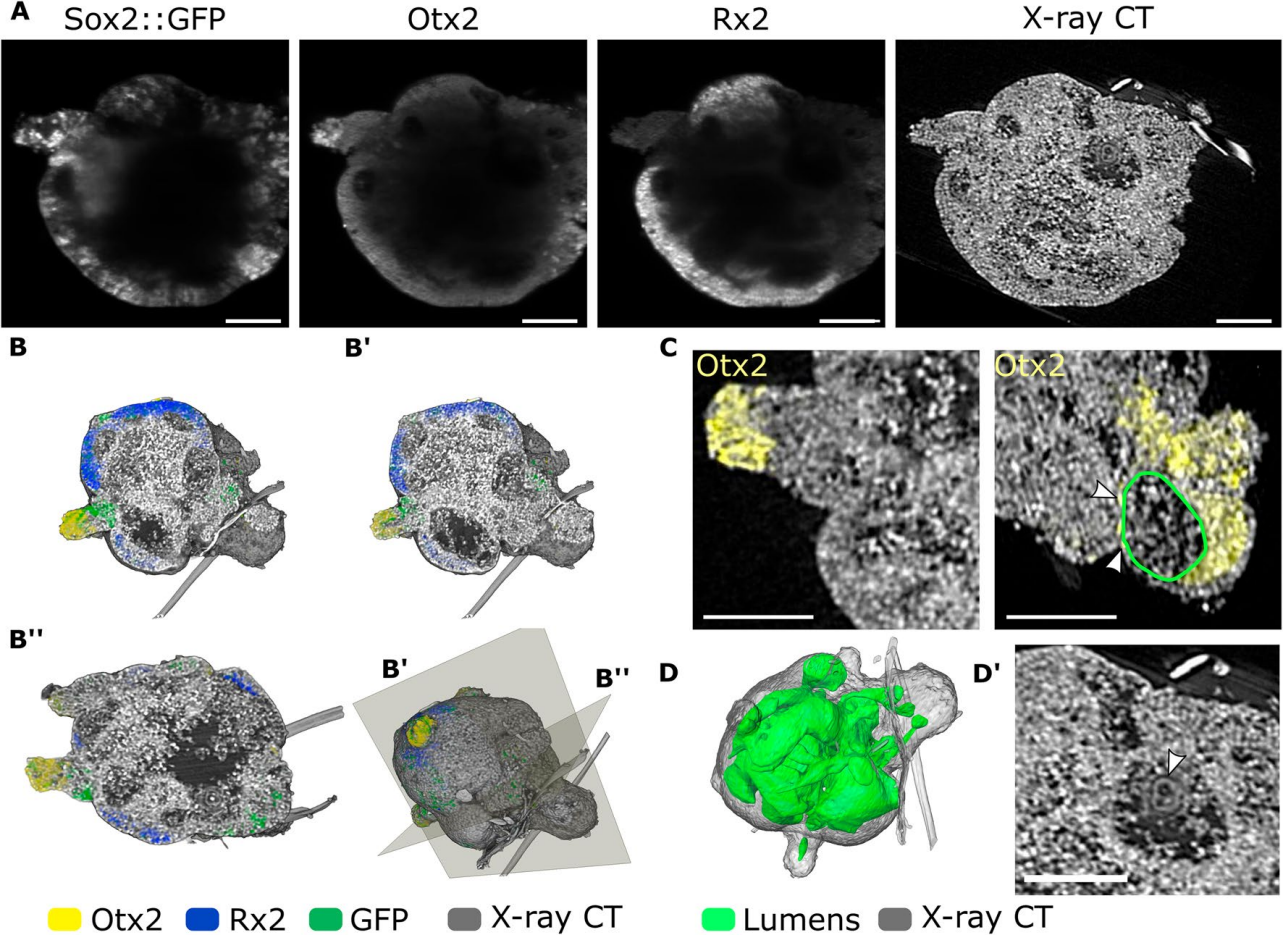
Complex structure of medaka-derived organoids revealed by multimodal imaging. (**A**) MuVi SPIM virtual slices of day 2 medaka-derived retinal organoids, generated from blastula stage *Sox2(600 bp)::GFP* transgenic embryos labelled with antibodies against GFP, Otx2 and Rx2 proteins. (**B**) 3D renderings of all contrast modalities seen in the direction of the volume behind the virtual cuts B′ and B′′ (the transverse and sagittal planes). (**C**) Otx2 expression in cells exhibiting different 3D shapes, the lumen is outlined in green. (**D**) The network of the lumen is shown in green within the organoid. (**D′**) Cellular content rendered based on X-ray tomography data within lumens is shown in panel (D). Scale bars are 100 µm.

We have previously reported on the ability of primary pluripotent cells of medaka to aggregate into retinal organoids, recapitulating the medaka-specific pace of development and mechanisms of cell migration during optic vesicle evagination^75^. Organoid cultures exhibit significant heterogeneity and variable complexity in cellular composition, calling for new approaches in imaging techniques^79,80^. We have performed multimodal imaging of fish retinal organoids generated from a transgenic reporter line *Sox2*(600 bp)::GFP in which GFP is expressed under the control of a *Sox2* regulatory element to monitor cells with neuronal progenitor identity (Supplementary Fig. 9). Co-staining of day 2 organoids with the antibodies against GFP, Rx2 and Otx2 proteins (Fig. 5) showed that most of the organoid tissue adopted retinal fate with a thick layer of neuroepithelial cells positive for general neuronal marker Sox2 with subdivision to non-retinal and retinal domains based on Rx2 expression (Fig. 5A, B). These non-retinal domains (Rx2^-^) overlap with the expression of the forebrain marker Otx2, suggesting that Rx2^-^ cells adopted anterior neuronal identity as described previously^75^. Similar separation of Sox2 and Otx2 domains are observed in the *Sox2(600 bp*)::GFP medaka embryos at stage 28 (Supplementary Fig. 10)*.* In organoids, we have found several domains of Otx2-positive cells with distinct flat morphology. From X-ray tomography, these cells surround the lumen within the retinal organoid and resemble the retinal pigmented epithelial layer seen in medaka embryos (Fig. 5C and Supplementary Fig. 10). Several studies have suggested that lumen plays an active part in tissue morphogenesis^81–83^. The complex spatial organisation of the lumen in the fish retinal organoid is clearly visible in the X-ray tomography data (Fig. 5D). Though not seen in fluorescence microscopy, the lumen is packed with cellular material, including complex membrane compartments (Fig. 5D′). Note, that we also found 3 strands integrated into the structure of the organoid (Fig. 5B and D). Their low X-ray absorption suggests that they are strands of plastics—a possible contamination from dishes, tips or environment. Visualisation of non-biological material along all cells and extracellular matrix in combination with gene expression patterns might provide an indispensable tool for 3D organoid cultures based on engineered approaches, where cells or cell aggregates are surrounded by biomaterials^84^.

## Discussions

Understanding cellular activity on the molecular level and within the biological context is essential for developmental biology and biomedical research. Previously, to put distinct molecular content back into the context of the surrounding tissue, a combination of bright-field contrast in light-sheet microscopy was exploited by other groups for in vivo tomographic reconstructions of overall morphology^14^. While providing the overall shape of the model organism, higher resolution and tissue sensitivity would significantly empower such correlative imaging approaches. We, therefore combined multiview light sheet fluorescence microscopy with X-ray computed tomography to enable high-resolution 3D imaging of expressed genes in healthy and mutant organisms and in in vitro systems.

Optical imaging modalities such as light sheet fluorescence microscopy are ideal for targeted visualisation at the molecular level, for example, of expressed genes and DNA/RNA sequences. However, the opacity of biological tissues limits 3D information obtained with fluorescence microscopy to several hundred micrometre-thick specimens. While this information depth was sufficient to analyse the expression of genes in small vertebrate model organism as medaka embryos, the visualisation of larger specimens need further development. To enable imaging of mm thick tissues protocols for clearing of tissues are currently under constant development^6,85^. Clearing protocols based on matching of refractive index, where tissue structure remains close to its native state, will be most promising for multimodal imaging with X-ray tomography.

Compared to other modalities, X-ray tomography offers high spatial resolution and differential contrast of tissues, acting as a hub for histology-like analysis. Through contrast-enhancing sample preparation, X-ray tomography has been used at the synchrotron radiation facilities and with laboratory X-ray tomography scanners to quantitatively assess the morphology of organs, tissues and cell densities within varying model organisms and biological tissues^22,23,40^. Similarly, a high differential contrast of tissues and cell nuclei was achieved in our multimodal imaging of medaka embryos and fish retinal organoids. To avoid shrinkage of tissues due to staining procedure water-based, contrast agents like iodine^40^ or ionic liquids^86^ can be used for sample preparation. The most promising further development is a combination of light-sheet microscopy, instead of absorption, with phase-contrast X-ray tomography^19,31^. Apart from simplified correlation and low radiation dose of phase contrast X-ray imaging, the combination of these techniques into a single instrument promises to enable in vivo multimodal imaging of biological systems^87^.

The whole protocol from sample preparation to registration takes 12 workdays with immunohistochemistry, SPIM imaging and registration as the most time-consuming steps. Although the timing of IHC cannot be significantly changed, higher numbers of samples can be processed simultaneously. The SPIM imaging can be shortened by using other SPIM geometries which enable simultaneous positioning and high-throughput imaging of several samples. Furthermore, if samples are oriented uniformly, alternative methods of registrations, such as ViBE-Z^52^ or CMTK^51^ potentially could reduce time required for registration.

Our work approaches the ideal of molecular and structural quantitative analysis in 3D for tissue samples and model organisms up to several hundred micrometres in size. The use of X-ray tomography to provide tissue context to the molecular pattern requires no modifications of the existing microscopes, relies on the right order for sample preparation and imaging, and uses existing open-source software to correlate two modalities. Multimodal acquisition provides a complete picture of the composition and molecular identity of tissues, which is especially important when the expression of fluorescence reporters is distributed in different anatomical regions. The ability to track and orient the expression of genes in specific tissues and perform quantitative analysis of their distribution is indispensable for the comparison of multiple samples with abnormal development and morphological defects. The technique is particularly suited to studying emerging model and non-model organisms and in vitro 3D tissue models.

## Materials and methods

### Fish stocks and husbandry

Medaka (*Oryzias latipes*) fish were maintained according to the local animal welfare standards (Tierschutzgesetz §11, Abs. 1, Nr. 1, husbandry permit AZ35-9185.64/BH, line generation permit number 35-9185.81/G-145/15 Wittbrodt). The following lines were used: Cab strain^88^ as a wild type and *Rx3*^*saGFP*^ line^75^.

The *Sox2(600 bp)::GFP* reporter line was generated for this study as following. The 600nt *Sox2* regulatory element was amplified via PCR from medaka Cab gDNA (forward primer (5′–3′): GAAATAGTAAATATGTACTTAGTATT, reverse primer (5′–3′): CAGGGAAAATTTTAACTTTTCGCTGGG). Plasmid ISceI/MCS-d1GFP-SV40/ISceI was digested with XhoI (NEB) and SmaI (NEB). The *Sox2(600 bp)::GFP* plasmid was generated by digesting the primer overhangs of the PCR product with XhoI and EcoRV (NEB) and its consequent ligation into linearized plasmid ISceI/MCS-d1GFP-SV40/ISceI. Injected embryos were raised and maintained at 26 °C followed by screening for GFP expression at stages 20–24 on a Nikon SMZ18.

### Fish retinal organoids

The fish retinal organoids were generated as described previously^75^. In short, blastula-stage (6 h post fertilisation) embryos^53^ were collected, dechorionated using hatching enzyme, and washed in ERM (17 mM NaCl, 40 mM KCl, 0.27 mM CaCl_2_, 0.66 mM MgSO_4_, 17 mM HEPES). The cell mass was separated from the yolk and transferred to PBS. After washing twice with PBS, the cell mass was dissociated by gentle pipetting with a 200 μl pipet tip. The cell suspension was pelleted (180 × g for 3 min), re-suspended in retinal differentiation media (GMEM Glasgow’s Minimal Essential Medium, Gibco Cat#:11,710,035), 5% KSR (Gibco Cat#:10,828,028), 0.1 mM non-essential amino acids, sodium pyruvate, 0.1 mM β-mercaptoethanol, 50 U/ml penicillin–streptomycin) and seeded into 96-well plates. The aggregates were incubated overnight at 26 °C in an incubator without CO_2_ control. The following day (day 1) aggregates were washed with retinal differentiation media, transferred to fresh wells, and Matrigel (Corning, Cat#:356,238) was added to the media to a final concentration of 2%. From day 1 onward, aggregates were incubated at 26 °C and 5% CO_2_.

### Whole-mount immunohistochemistry

Whole-mount immunohistochemistry was performed as described previously^89^ with modifications.

Embryos were sorted according to stages 18–33^53^ and fixed overnight in 4%PFA in PTW (PBS with 0.05% Tween-20) at 4 °C. After fixation, embryos were washed with PTW. Embryos with pigmented retinae were bleached with 3% H2O2, and 0.5% KOH in PTW in the dark. Samples were heated in 50 mM Tris–HCl for 15 min at 70 °C, permeabilized with precooled acetone for 15 min at − 20 °C and incubated in the blocking solution (1% BSA, 1% DMSO, 4% sheep serum) overnight at 4 °C. Samples were incubated with primary antibody (1:200) in the blocking buffer for two days at 4 °C and washed with PTW 5 times 10 min. The following antibodies were used: anti-phospho-histone H3 (rabbit, Millipore, Cat#: 06–570), anti-HuC/D (mouse, Thermo Fisher Scientific, Cat.#: A21271), anti-Otx2 (goat, R&D systems, Cat.#: AF1979), anti-N Cadherin (rabbit, abcam, Cat.#: ab76011). Samples were incubated with secondary antibody (1:750) and DAPI (1:500) or DRAQ5 (Thermo Fisher Scientific, Cat#: 65-0880-92; 1:1000) nuclear stain in the blocking buffer for 2 days at 4 °C and washed with PTW 3 times 10 min.

Day 2 organoids were fixed overnight in 4% PFA in PTW at 4 °C. Samples were washed in PTW, heated in 50 mM Tris–HCl at 70 °C for 15 min, permeabilized for 15 min in acetone at − 20 °C, and blocked in 10% BSA in PTW for 2 h. Samples were incubated with primary antibody (1:500) for 3 days at 4 °C. Following antibodies were used: rabbit anti-Rx2 (rabbit, in-house^76^, chicken anti-GFP (Thermo Fisher Scientific, Cat#: A10262), goat anti-Otx2 (R&D Systems, Cat#: AF1979). Samples were washed with PTW and incubated secondary antibody (1:750) overnight at 4 °C. At the end, samples were washed with PTW and mounted for imaging.

### Fluorescent in-situ hybridisation

Embryos were sorted by stages^53^ and fixed in 4% PFA in 2 × PTW overnight at 4 °C. Fluorescent whole mount in situ hybridizations were performed as described previously^90^. In brief, samples were rehydrated in the reverse series of MeOH and washed twice for 15 min with PTW. Afterwards, they were digested with 10 µg/ml proteinase K in PTW at room temperature without shaking for the following periods: st.22–23–9 min, st.28–15 min, st.33–75 min. To stop the digestion, samples were rinsed twice in 2 mg/ml glycine in PTW. An additional fixation step was carried out with 4% PFA in PTW for 20 min at room temperature while gently shaken, followed by 5 times for 5 min washing steps in PTW. For the pre-hybridisation, samples were incubated with a hybridization mix (50% formamide, 5 × SSC, 50 µg/ml heparin, 0.1% Tween20, 5 mg/ml torula RNA) at 65 °C for 2 h. For the probe mix preparation, 10 µl of digoxigenin-labelled *pax6* (ENSORLG00000009913) riboprobe was mixed with 200 µl of the hybridization mix and denatured at 80 °C for 10 min. After pre-hybridization, the hybridization mix was replaced by the probe mix. Samples were hybridised at 65 °C overnight. Then the probe mix was removed and samples were washed 2 × 30 min with 50% formamide in 2 × SSCT at 65 °C, followed by a washing step in 2 × SSCT for 15 min at 65 °C, and 2X in 0.2 × SSCT for 30 min at 65 °C. The following 3 washing steps in TNT (0.1 M Tris pH 7.5, 0.15 M NaCl, 0.1% Tween20) were performed at RT for 5 min. Blocking was performed by incubating samples in TNB (TNT with 2% blocking reagent) on a rotator for 2 h at RT. Anti-Dig-POD antibody (1:100) and primary antibody (anti-Otx2, R&D Systems, Cat#: AF1979) in 100 µl of TNB were added to samples. Samples were incubated rotating at 4 °C overnight and subsequently were washed 5 × 10 min with TNT and rinsed with 100 µl TSA Amplification Diluent. Cy3 Fluorophore Tyramide was diluted 1:50 in TSA Amplification Diluent, and 100 µl of the staining solution was added to the samples. Samples were stained for 2 h at RT without shaking. After the staining, samples were washed 3 × 10 min with TNT and incubated with the secondary antibody and DAPI nuclear stain in TNT at 4 °C overnight. Afterwards, the samples were washed and imaged with a light-sheet microscope.

### Light-sheet microscopy

3D fluorescent imaging of all specimens was performed on the 16 × detection MuVi SPIM Multiview light-sheet microscope (Luxendo Light-sheet, Bruker Corporation). To ensure that the specimens can be used for further imaging with X-rays, instead of mounting specimens with 2% low melting agarose, they were rested on the 2% agarose as shown in Supplementary Fig. 1. After solidification of the agarose, the tube was further filled with 1 × PBS and samples were pipetted inside the tube. The tube was placed vertically such that the specimen fell onto the solid support of agarose.

Two volumes at 0 and 90 rotation each, with 1.6 µm z step size for three channels, that is, GFP 488 nm excitation laser at 80% intensity, 525/50 nm emission filter, 200 ms exposure time, RFP 561 nm excitation laser at 30% intensity, 607/70 nm emission filter, 100 ms exposure time, and far-red 642 nm excitation laser at 50% intensity, 579/41 nm emission filter, 250 ms exposure time, were acquired. The four volumes for each channel were then combined with the Luxendo Light-Sheet microscopy software.

### Synchrotron X-ray microtomography

To improve X-ray absorption of soft tissues, samples were dehydrated in ethanol series of 10%, 30%, 50% and 70% for 1 h each and stained with 0.3% phosphotungstic acid in 70% ethanol, respectively, for 1 day at room temperature. After the staining procedure, the samples were washed in 70% ethanol and sealed in polypropylene containers for X-ray tomography.

All specimens were scanned at the IPS UFO 1 tomographic station at the Imaging Cluster of the KIT Light Source. A parallel polychromatic X-ray beam produced by a 1.5 T bending magnet was spectrally filtered by 0.5 mm aluminium to remove low-energy components from the beam. The resulting spectrum had a peak at about 17 keV, and a full-width at half maximum bandwidth of about 10 keV. X-ray projections were detected by a CMOS camera (pco.dimax, 2016 × 2016 pixels, 11 × 11 µm^2^ pixel size) coupled with an optical light microscope (Optique Peter; total magnification 10 ×). Photons were converted to the visible light spectrum by an LSO: Tb scintillator of 13 µm. A complete optical system resulted in an effective pixel size of 1.22 µm. For each specimen, a set of 3000 projections with 70 ms exposure time was recorded over a 180° tomographic rotation axis. The 3D volumes were reconstructed using the filtered back projection algorithm implemented in tofu^91^.

### Correlation of light-sheet and X-ray microtomography

For the correlation of two modalities in 3D, it is important to find a transformation matrix which would include all degrees of freedom. Because the MuVi SPIM dataset is larger (higher spatial resolution and up to 3 fluorescent channels), the X-ray tomography volume is registered to MuVi SPIM. Before the process of registration, the X-ray tomography volume was compressed from 32-bit to 8-bit depth. Similarly, MuVi SPIM volumes were binned to match the spatial resolution of the X-ray tomography (from 400 nm to 1.22 µm) and converted to 8-bit images. To improve registration of sparse labels all channels of MuVi SPIM were summed up and used for further correlation.

To find the transformation matrix between X-ray tomography and MuVi SPIM, we used the fijiyama plugin designed to correlate datasets from different modalities^48^. In short, for rigid transformation, two volumes were rotated and oriented manually, followed by similarities transformation based on points of interest selected manually in two datatypes as shown in Supplementary Fig. 2, that is lenses, ears, tail and frontal part of the head. The manual registration was followed by automatic block-matching (number of iterations 20) with similarities, followed by a dense vector field. After the final registration step, all transformation matrixes were merged into one and applied to the original quality (uncompressed) X-ray tomography and light-sheet data.

### Segmentation, image analysis and plotting

Segmentation of retinas, brain and lenses was performed in Dragonfly software (Version 2020.2 for Windows, Object Research Systems (ORS) Inc, Montreal, Canada, 2020; software available at http://www.theobjects.com/dragonfly). The fluorescent signal from all SPIM channels was segmented by automatic Otsu threshold using combined retina and brain labels. The resulting mask was then intersected with either the retina or brain label. The values were normalized by the voxel size of the corresponding label. The plots were prepared with RStudio (RStudio Team (2020). RStudio: Integrated Development for R. RStudio, PBC, Boston, MA URL http://www.rstudio.com/) using packages ggplot2^92^ https://cran.r-project.org/web/packages/ggplot2/citation.html, patchwork (Pedersen TL., 2023) https://patchwork.data-imaginist.com/authors.html, ggpattern (FC M, Davis T., 2022) https://coolbutuseless.github.io/package/ggpattern/authors.html, tidyr (Wickham H, Vaughan D, Girlich M 2023) https://tidyr.tidyverse.org/authors.html, grid (Murrell P, 2005) https://stat.ethz.ch/R-manual/R-devel/library/grid/html/grid-package.html. Figures were assembled using Affinity Designer 2 and Inkscape.

### Supplementary Information

Supplementary Information 1.

Supplementary Video 1.

Supplementary Video 2.

Supplementary Video 3.

## Data Availability

Data is provided within the manuscript or supplementary information files.
