# Supplementary material for: Visualisation of gene expression within the context of tissues using an X-ray computed tomography-based multimodal approach
Source: Sci Rep. 2024 Apr 12;14:8543. doi: 10.1038/s41598-024-58766-5 (PMC11015006; doi:10.1038/s41598-024-58766-5)
Supplement: Supplementary file 1 — Supplementary Information 1. [file 41598_2024_58766_MOESM1_ESM.pdf]

Supplementary information

Visualisation of gene expression within the context of tissues using an X-ray computed tomography-based multimodal approach

Kristaps Kairišs[1,2,†], Natalia Sokolova[1,3,†], Lucie Zilova[1], Christina Schlagheck[1,2,3], Robert Reinhardt[1,6], Tilo Baumbach[4,5], Tomáš Faragó[4], Thomas van de Kamp[4,5], Joachim Wittbrodt[1], and Venera Weinhardt[1]*

[1] Centre for Organismal Studies, 69120 Heidelberg, Germany

[2] HeiKa Graduate School on "Functional Materials", Heidelberg, Germany

[3] Heidelberg International Biosciences Graduate School HBIGS, Heidelberg, Germany

[4] Institute for Photon Science and Synchrotron Radiation (IPS), Karlsruhe Institute of Technology (KIT), Eggenstein-Leopoldshafen, Germany

[5] Laboratory for Applications of Synchrotron Radiation (LAS), Karlsruhe Institute of Technology (KIT), Karlsruhe, Germany

[6] European Molecular Biology Laboratory, Heidelberg, Germany

[†]Contributed equally

*For correspondence: venera.weinhardt@cos.uni-heidelberg.de

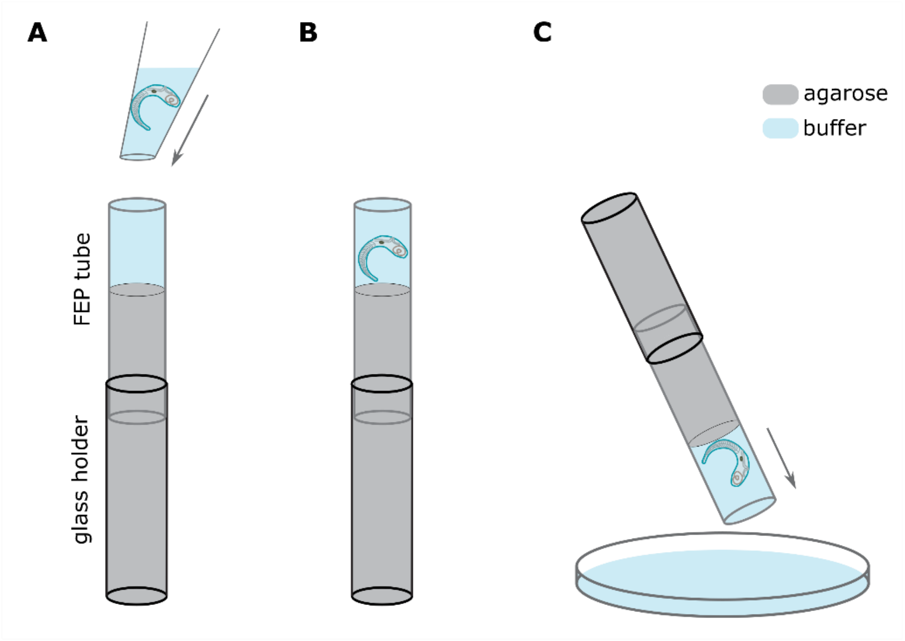

Supplementary figure 1 Embedding of specimens for light-sheet microscopy to enable multimodal pipeline. (A) A sample holder for MuVi SPIM consisting of the glass tube and a Fluorinated ethylene propylene (FEP) tube is filled with 2% agarose (in grey) and buffer (in blue). The specimen is pipetted on top with the large opening pipette tip. (B) Due to gravitation, the sample will sink to the bottom of the buffer-filled region, the process can be sped up by gently tapping the tube on the table. (C) The sample is removed by flipping the MuVi SPIM holder into a petri dish filled with buffer. To ensure that the sample is not moving during the acquisition, it is important to select the diameter of the FEP tube matching the gross

morphology of the specimen or create a conically shaped agarose mould as previously described (Moosmann et al. 2014).

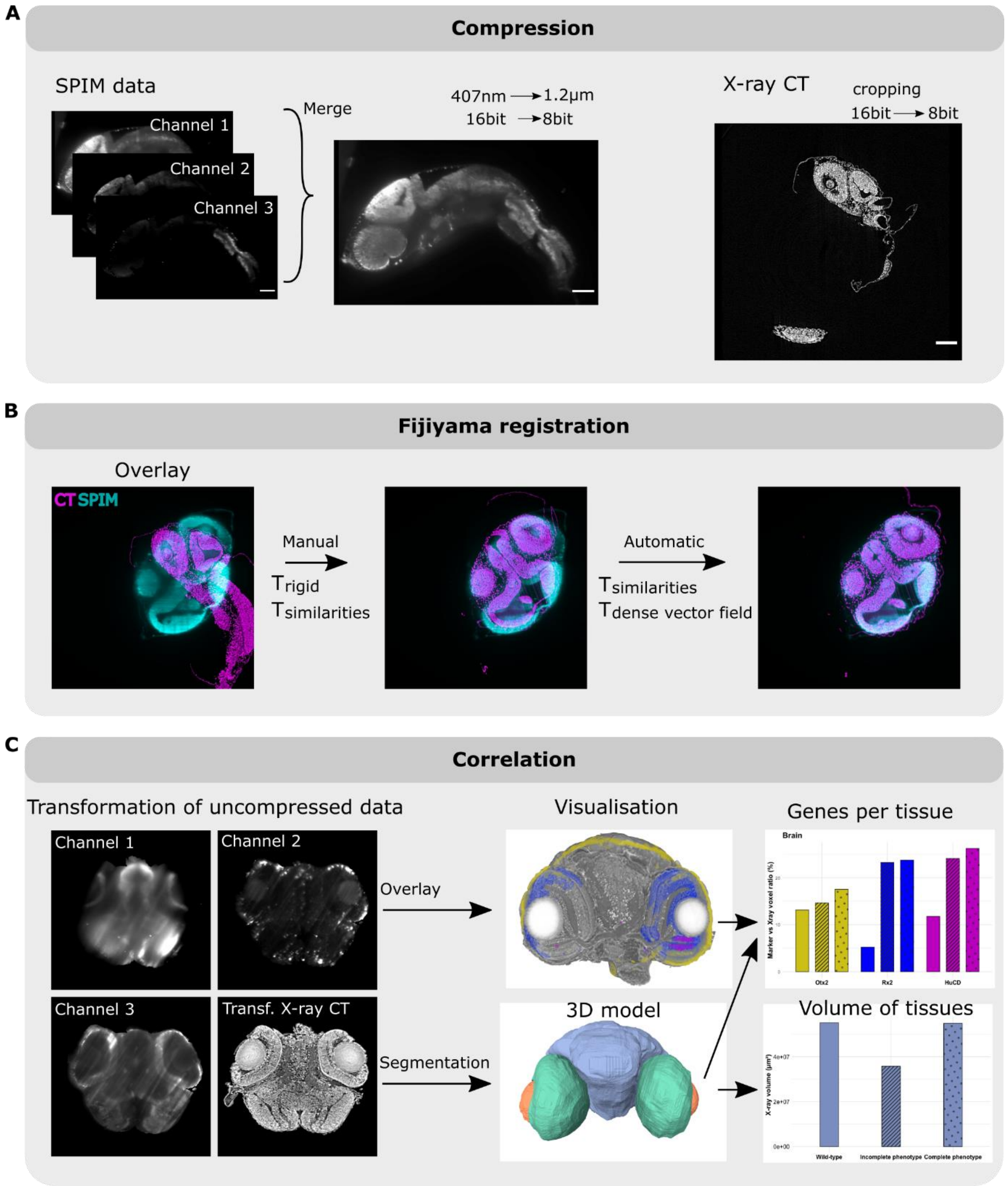

Supplementary figure 2 Correlation and analysis of light-sheet microscopy and X-ray microtomography datasets. (A) The first step in a correlation pipeline is compression. All fluorescent signals of MuVi SPIM are merged in one dataset, rescaled to the resolution of X-ray tomography (here 1.2 µm) and compressed to 8-bit. The X-ray tomography is cropped and compressed to 8-bit. (B) The datasets are correlated with the open-source plugin Fijiyama (Fernandez and Moisy 2021). In brief, two datasets are manually oriented to match the overall orientation of the specimen and then rigid transformation is performed on the X-ray tomography dataset. This manual alignment is followed by an automatic block-matching approach with similarities transformation, which would compensate for shrinkage due to dehydration in contrast to deposition for X-ray tomography. Two more automatic registrations

are then performed with similarities and dense vector field transformation, resulting in a transformation matrix of X-ray to MuVi SPIM dataset. (C) The transformation matrix is applied to the original X-ray tomography dataset and overlayed with uncompressed individual channels. Based on the tissue structure visible in X-ray tomography, segmentation and quantitative evaluation of tissue morphology and corresponding gene expression are performed. Scale bars are 100 µm.

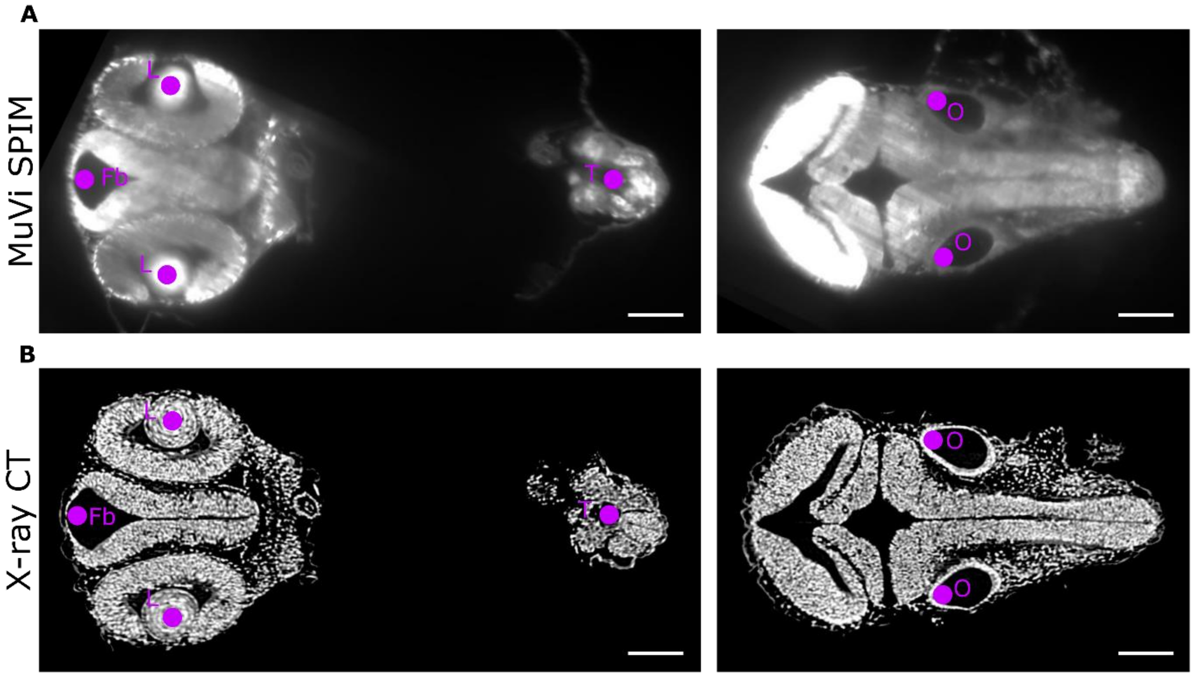

Supplementary figure 3 Identification of anatomical features for manual rigid registration. Coronal planes through dataset acquired with the MuVi SPIM (A) and X-ray microtomography (B). A minimum of 5 points are required for rigid registration between datasets. In medaka embryos lenses (L) and otoliths (O) are supplemented by the most anterior part of the brain (Fb) and the most posterior part of the tail (T), specifically the neuronal tube. Scale bars are 100 µm.

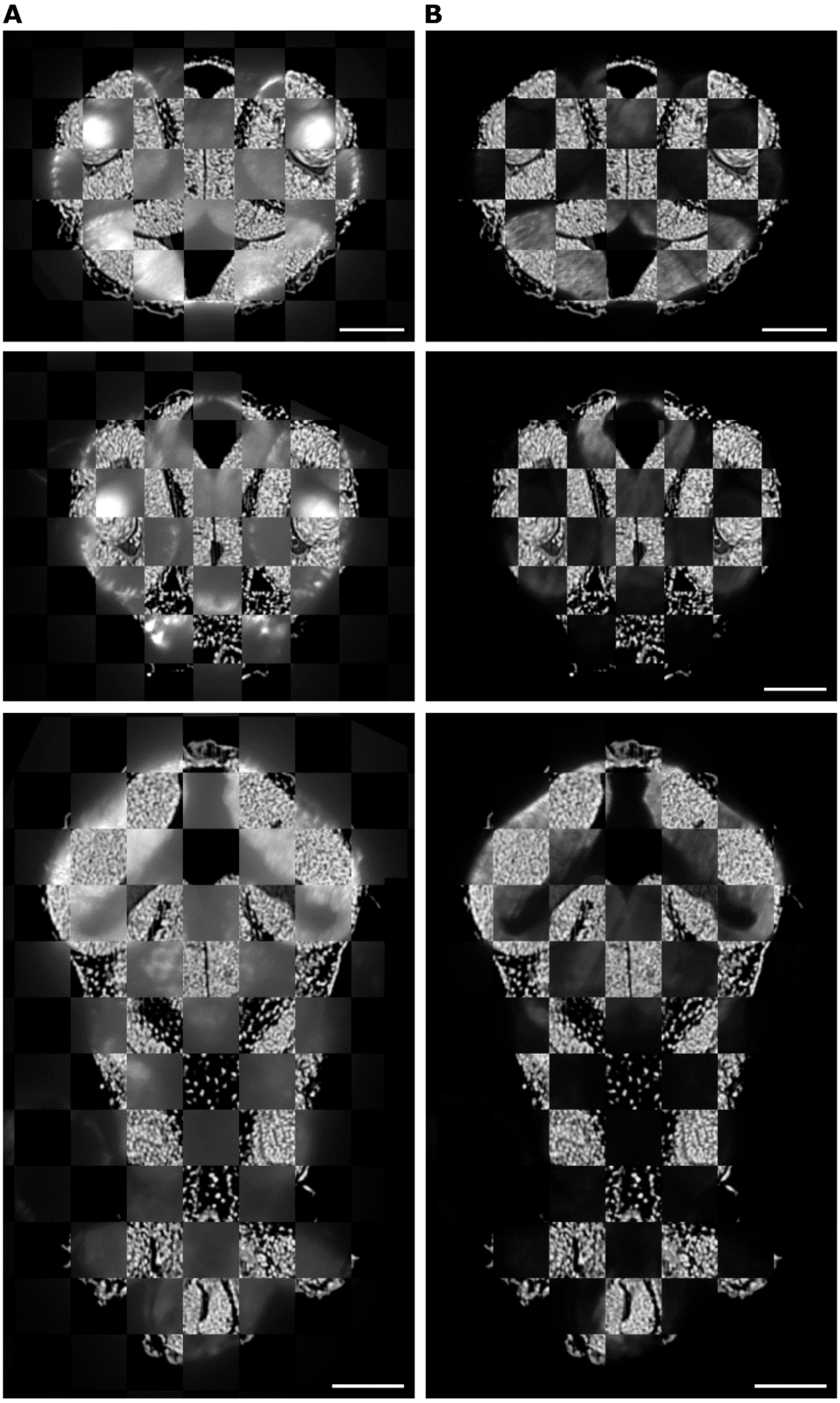

Supplementary figure 4 Checkerboard visualization facilitates visual evaluation of the registration accuracy. Overlay of X-ray tomography and MuVi SPIM green (A) and far-red (B) fluorescent channels at three coronal sections through medaka embryo at stage 28. Scale bars are 100 µm.

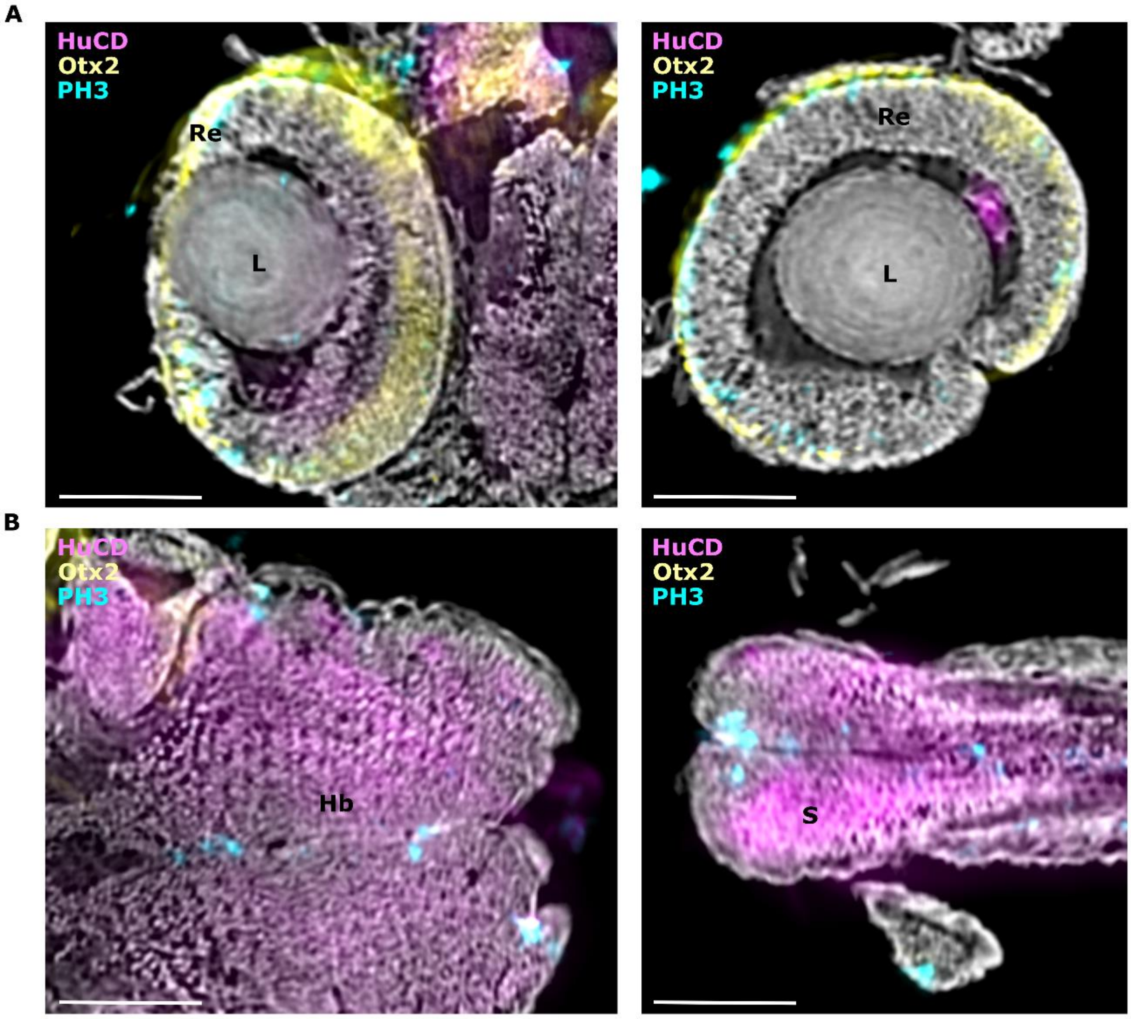

Supplementary figure 5 (A) Virtual slices through eye region in multimodal imaging on stage 24 of medaka embryo, shown in Figure 2. (B) Virtual slices through the hindbrain and somite of multimodal imaging on stage 24 of the medaka embryo, are shown in Figure 2. Organs are labelled as follows: lenses (L), retina (Re), hindbrain (Hb), and somite (S). Scale bars are 100 µm.

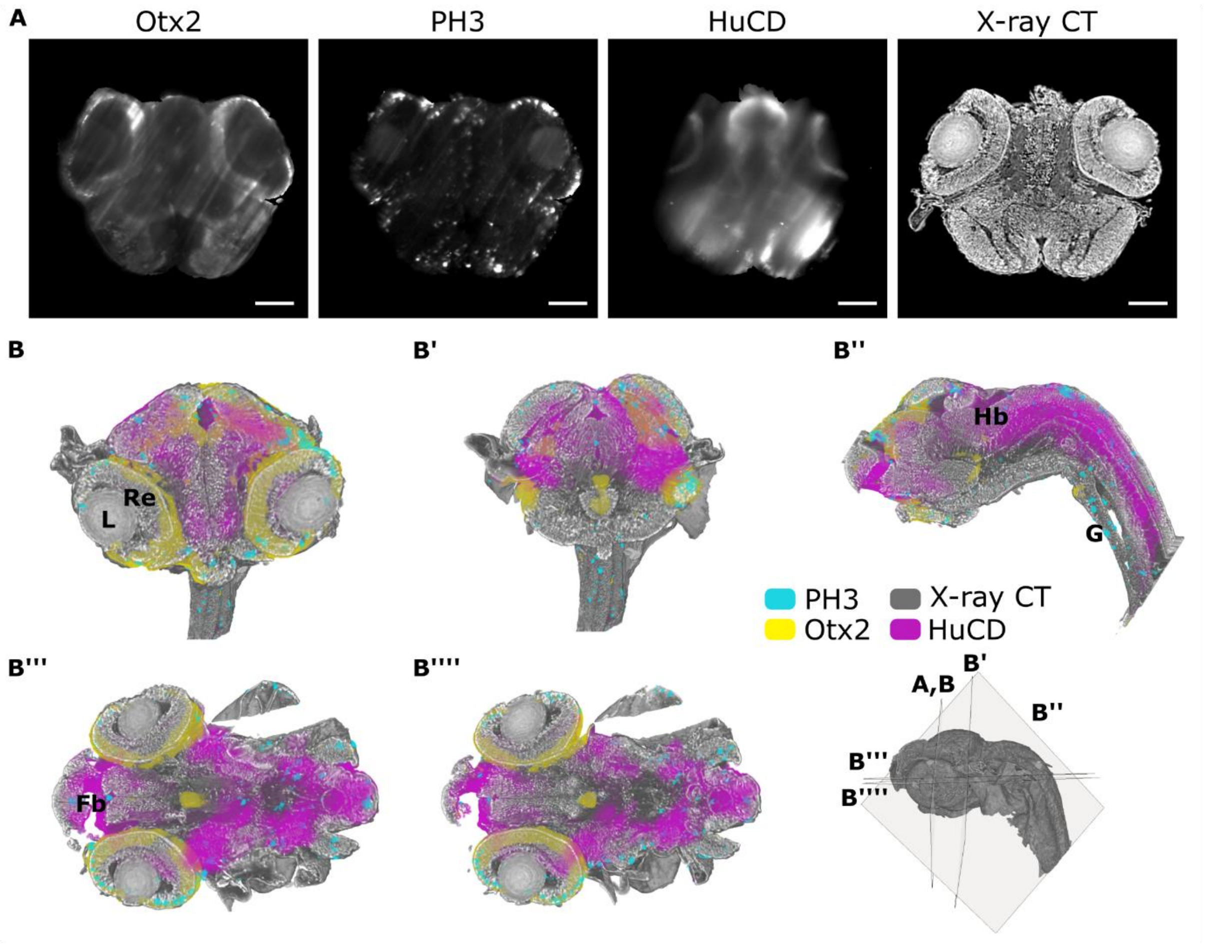

Supplementary figure 6 (A) Virtual sagittal slices of registered MuVi SPIM and X-ray tomography datasets. From left to right immunolabelling of Otx2, PH3 and HuC/D along with X-ray absorption in stage 32 of medaka embryo. (B) 3D renderings of all contrast modalities visualized with virtual cuts in axial, coronal and sagittal planes. The position and orientation of cuts is shown in the 3D rendering of the whole embryo. Organs are labelled as follows: lenses (L), retina (Re), forebrain (Fb), hindbrain (Hb), gut tube (G) and somite (S). Scale bars are 100 µm.

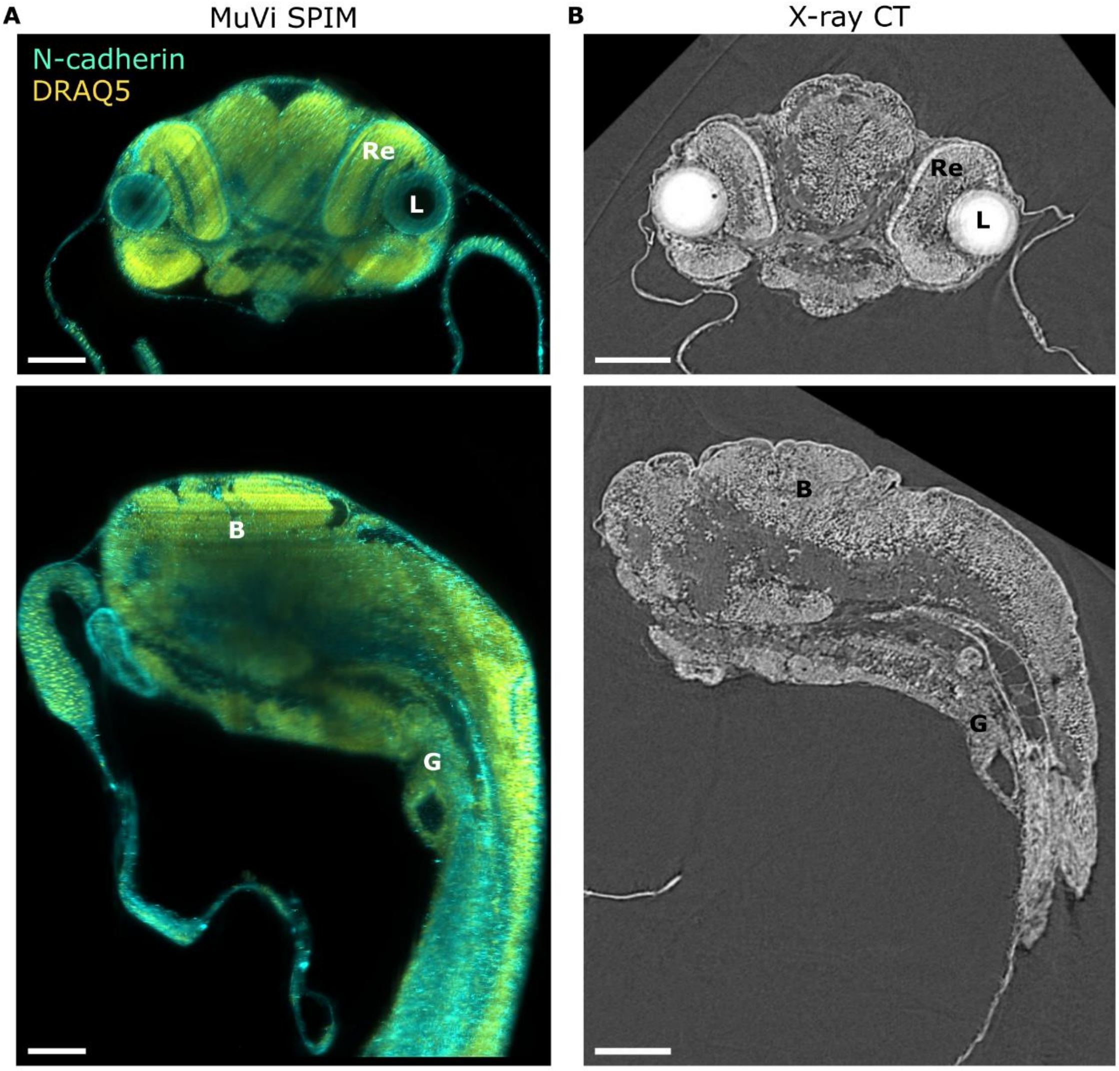

Supplementary figure 7 Difference in tissue contrast between fluorescence and X-ray contrast in medaka embryos. (A) Transverse and sagittal virtual slices of medaka embryo (stage 24) stained with antibody against transmembrane protein N-cadherin, co-labelled with nuclear stain DRAQ5 and imaged with the MuVi SPIM. (B) The same as in panel (A) specimen and virtual slices stained with phosphotungstic acid imaged with X-ray tomography. Organs are labelled as follows: lens (L), retina (Re), brain (B) and gut tube (G). Scale bars are 100 µm.

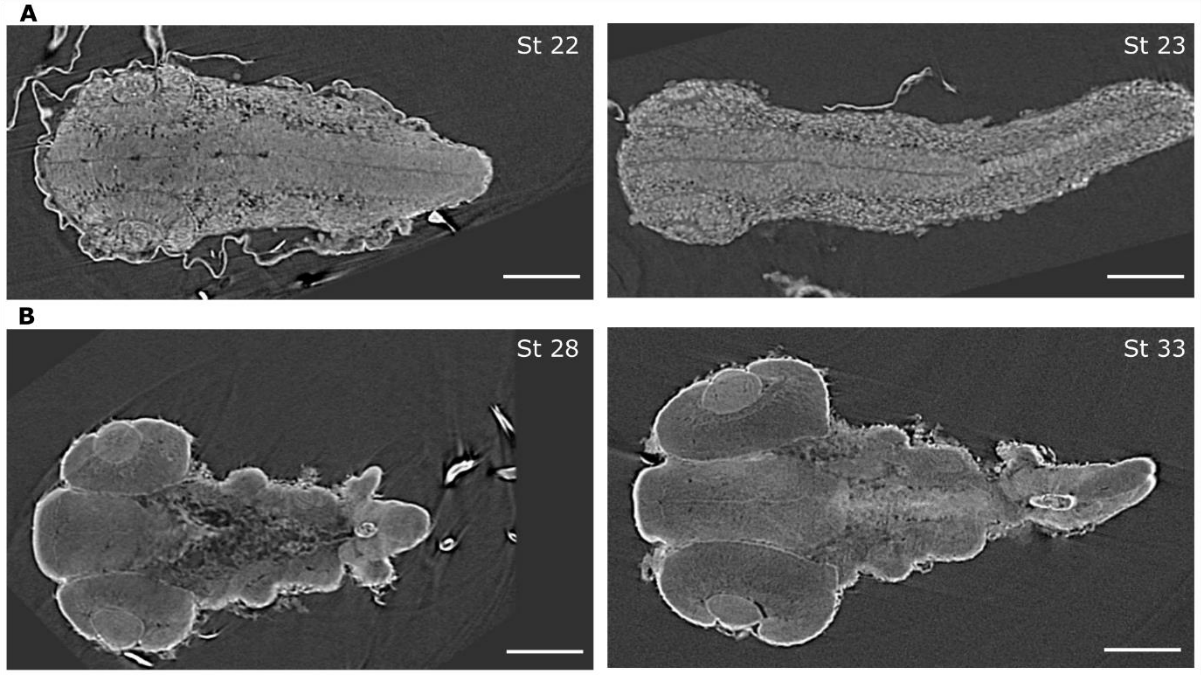

Supplementary figure 8 Differences in PTA staining after RNA *in situ* hybridisation for different stages of medaka embryo. (A) Stages 22 and 23 and (B) stages 28 and 33 of medaka embryos with phosphotungstic acid staining after fluorescence *in situ* hybridisation. All specimens were stained with the same reagents and under the same conditions. Scale bars are 100 µm.

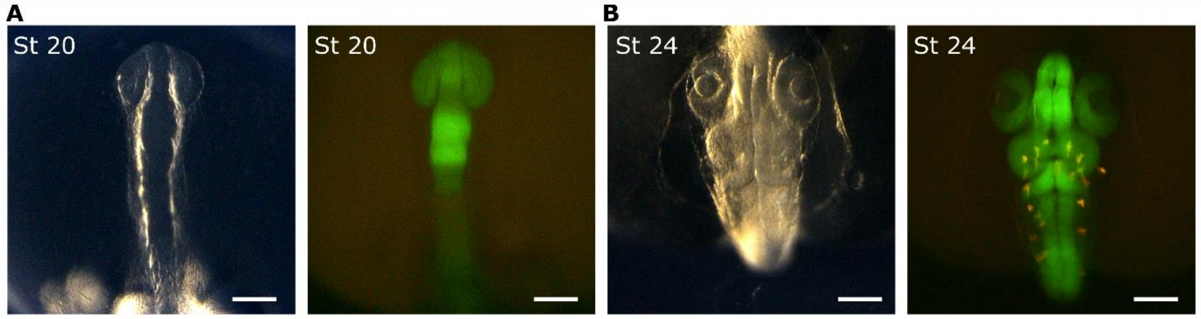

Supplementary figure 9 Characterization of *Sox2(600bp)::GFP line*. Bright-field and fluorescence images of *Sox2(600bp)::GFP line* and corresponding sox2 domain of GFP in the developing embryos at stages 20 and 24. Scale bars are 100 µm.

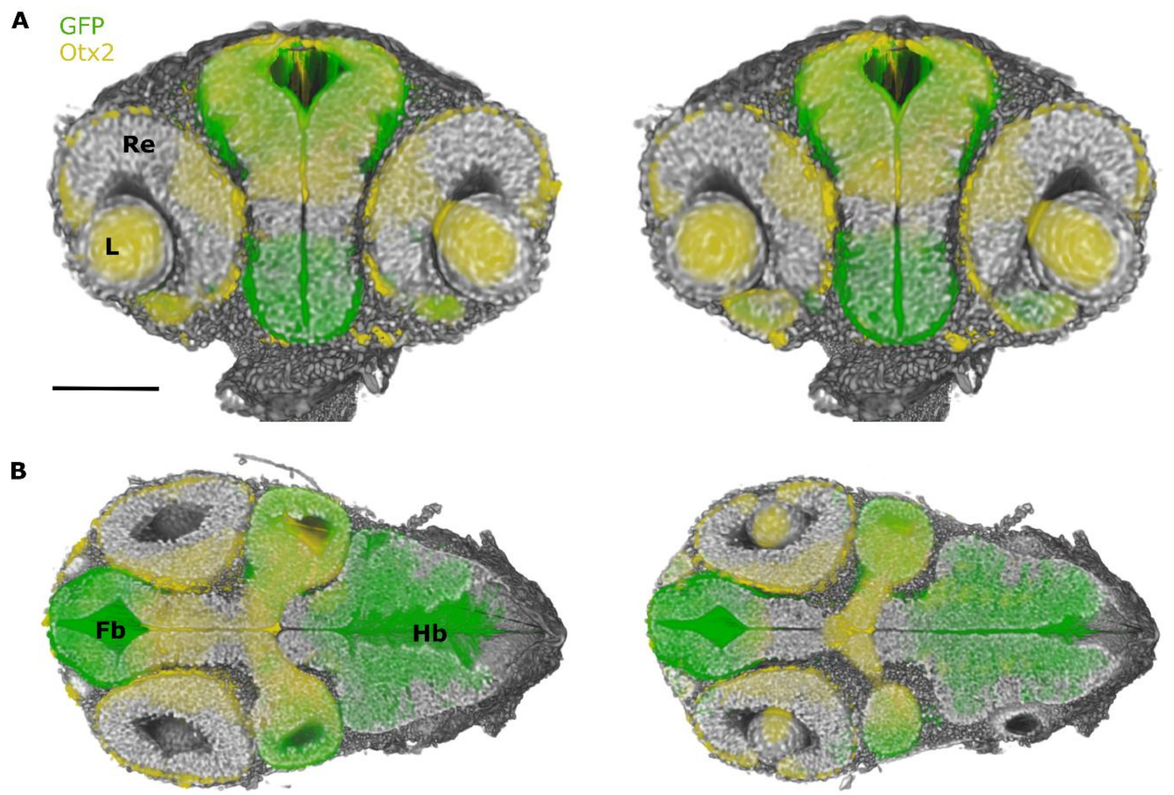

Supplementary figure 10 Characterization of *Sox2(600bp)::GFP line* at stage 28 by multimodal approach. (A) Retinal domains outlined by Otx2 expression only and non-retinal neuronal domains expressing *sox2* and Otx2 in axial cuts along the eye and eye with optical nerve. (B) Specificity of *sox2* expression to the neuronal non-retinal domain in coronal sections of the brain region. Organs are labelled as follows: lenses (L), retina (Re), forebrain (Fb), and hindbrain (Hb). The scale bar is 100 µm.

Video legends

Video 1 Neuronal genes in the context of tissue anatomy of the medaka embryo stage 24 visualized by the multimodal approach. 3D rendering of all contrast modalities, see Figure 2, Otx2 in yellow, PH3 in cyan and HuC/D in magenda visualized with virtual cuts through the whole volume.

Video 2 Tissue architecture of phenotypic variations in *Rx3^{saGFP}* mutants based on X-ray tomography. 3D renderings of segmented tissues, that is brain (blue), retina (green), and lens (orange) in wild-type, incomplete and complete phenotypes.

Video 3 Complex 3D network of the lumen in medaka-derived organoids revealed by X-ray tomography. 3D rendering of organoid and segmented lumen presented as surface view in green within it, see also Figure 5D.
